# Cost-effectiveness of anti-retroviral therapy at a district hospital in southern Ethiopia

**DOI:** 10.1186/1478-7547-7-13

**Published:** 2009-07-17

**Authors:** Asfaw Demissie Bikilla, Degu Jerene, Bjarne Robberstad, Bernt Lindtjørn

**Affiliations:** 1 Center for International Health, University of Bergen, Post box 7804, 5020 Bergen, Norway; 2 Faculty of Business and Economics, Hawassa University, PO Box 278, Hawassa, Ethiopia; 3 Arba Minch Hospital, Arba Minch, Ethiopia; 4 Department of Public Health and Primary Health Care, University of Bergen, Bergen, Norway

## Abstract

**Background:**

As the resource implications of expanding anti-retroviral therapy (ART) are likely to be large, there is a need to explore its cost-effectiveness. So far, there is no such information available from Ethiopia.

**Objective:**

To assess the cost-effectiveness of ART for routine clinical practice in a district hospital setting in Ethiopia.

**Methods:**

We estimated the unit cost of HIV-related care from the 2004/5 fiscal year expenditure of Arba Minch Hospital in southern Ethiopia. We estimated outpatient and inpatient service use from HIV-infected patients who received care and treatment at the hospital between January 2003 and March 2006. We measured the health effect as life years gained (LYG) for patients receiving ART compared with those not receiving such treatment. The study adopted a health care provider perspective and included both direct and overhead costs. We used Markov model to estimate the lifetime costs, health benefits and cost-effectiveness of ART.

**Findings:**

ART yielded an undiscounted 9.4 years expected survival, and resulted in 7.1 extra LYG compared to patients not receiving ART. The lifetime incremental cost is US$2,215 and the undiscounted incremental cost per LYG is US$314. When discounted at 3%, the additional LYG decreases to 5.5 years and the incremental cost per LYG increases to US$325.

**Conclusion:**

The undiscounted and discounted incremental costs per LYG from introducing ART were less than the per capita GDP threshold at the base year. Thus, ART could be regarded as cost-effective in a district hospital setting in Ethiopia.

## Background

In 2003, some selected health institutions started to offer anti-retroviral therapy (ART) in Ethiopia. In 2005, a nationwide ART programme was launched and the service was decentralised to health centres in 2006 [1]. By March 2009, the number of treatment sites had reached 343, and 189,267 patients (56% of those in need) received ART [2]. According to the official Ministry of Health (MOH) reports, the projected adult HIV prevalence for the year 2009 was estimated at 2.3 %, with about 336,160 adults and 20,522 children being in need of ART [3].

Given the growing need for ART in Ethiopia, the resource implications of expanding the treatment is likely to be great. Therefore, it is necessary to consider whether providing ART is worth doing compared with treating and caring for HIV patients without ART, and if so, how much extra resources would be needed to treat an HIV patient with ART in Ethiopia. These questions have direct relevance to the further expansion of ART, and the answers may facilitate resource mobilization in the country. Thus, there is a need to know the cost and effectiveness of ART in routine clinical practice.

The evidence on the cost-effectiveness of ART from Africa, though still meagre, suggest that ART is a cost effective intervention in developing countries [4-6]. Two studies from South Africa [4,7] suggest that ART is cost-effective compared to no-ART. One of these [4] found, based on 2004 prices, an average per person year (PPY) cost of ART ranging from US$850 to US$1,645 with an incremental cost that range from being cost saving to US$1,772 per life year gained (LYG). The other study [7] reported, based on 2003 prices, an undiscounted and discounted incremental costs of US$1,023 and US$984 respectively per LYG. A study from Cote d'Ivoire, a low income country and therefore more comparable with Ethiopia [8], reported a discounted incremental cost of ART ranging from US$542 to US$829 per LYG based on 2002 prices [9]. A region-based study on ART by Hogan *et al. *[5] reported an incremental cost, in the year 2000's international dollars, ranging from $556 to $596 per disability-adjusted life years (DALYs) for sub-Saharan countries.

As illustrated above, the health economic evidence of ART in resource-constrained countries is limited. Much of what is available has also come from South Africa, which is an upper middle income country [8] and wealthier than typical sub-Saharan countries. The region-based studies also represent larger African regions and may not necessarily represent the conditions in individual countries. Specifically, there is no information from Ethiopia. Our study aims to assess the cost-effectiveness of ART in a routine clinical practice setting in southern Ethiopia.

## Methods

### Study setting

We carried out our study at an HIV clinic at Arba Minch Hospital (AMH) in the Southern Nation, Nationalities and Peoples' Region (SNNPR) in Ethiopia. The hospital started offering ART in August 2003, and follows national and World Health Organaization (WHO) recommendations [10-12] in the management of the program. The ART regimens for adults included the following combinations of first-line drugs: (Stavudine-Lamivudine-Nevirapine), (Zidovudine-Lamivudine-Nevirapine), (Stavudine-Lamivudine-Efavirenz) and (Zidovudine-Lamivudine-Efavirenz). The HIV clinic at AMH had one physician, one nurse, one data clerk and two community healthworkers. Those patients who were not on ART come to the clinic every three month for followup whereas those on ART come every month for monitoring and medication refill. Upon visit to the clinic, all HIV patients see a doctor. Further account of the setting is presented elswhere [13].

### Study design

Our study compares the costs and health outcomes of ART with the alternative scenario of treating and caring for HIV patients without ART (no-ART) for routine clinical practice at a district hospital. The comparator no-ART involves treatment of opportunistic infections and prophylaxis with cotrimoxazole, but no anti-retroviral drugs. The measure of health effect is LYG under no-ART and ART. The study adopts a health care provider perspective and focuses on hospital costs. We used a Markov life cycle model to analyse the lifetime cost and effects of the two treatment alternatives.

### Model characteristics

**Figure 1:**
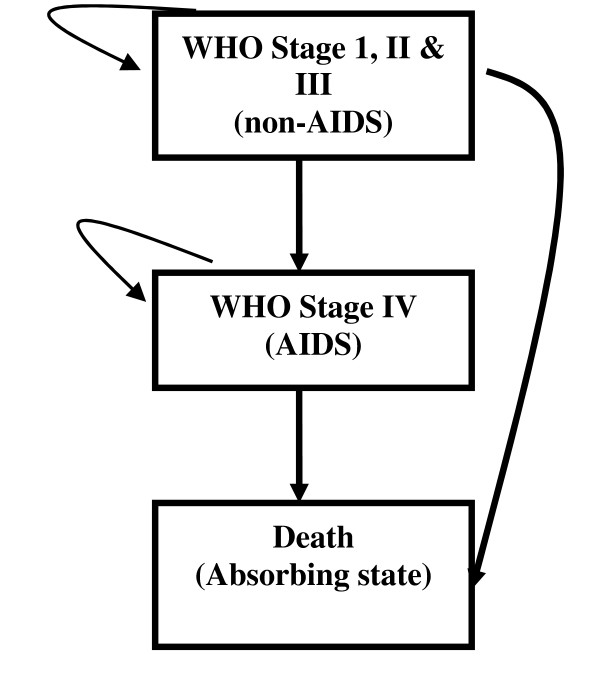
**The Markov states and pattern of HIV disease progression**.

Markov modelling is suitable for analysing different outcomes when the clinical course of the disease has an extended time horizon, and when the nature of the condition is such that patients experience different health states at different points in time. The technique allows estimating life expectancies and lifetime costs [14-16]. We based our model on the WHO HIV clinical staging system [12], grouping the four clinical stages into two health states: non-AIDS HIV state (i.e. WHO clinical stages I, II & III); and AIDS state (i.e. WHO stage IV). The two alive clinical states of the HIV disease (i.e. no-AIDS and AIDS states) and the 'dead' state (i.e. death from AIDS) form the three states in the Markov model of our study (Figure 1).

We set the length of the Markov cycle to three months; and populated the model with transition probabilities and average cycle costs. The transition probabilities are the risks of progressing to the next worst health state within a 3-month period, whereas the cycle cost is the average cost of service use associated with staying in each of the Markov states in a 3-month period. Then patients in each of the comparators and respective AIDS states were evaluated at the end of each cycle (3 month) to determine whether they remained in their current state or had moved to the next worse state. We repeated this, through cohort simulation, for several cycles over an extended period of time to estimate life expectancy and life time cost. We used beta distribution for the transition probabilities and gamma distribution for the costs during the analysis.

We derived the model input parameters (three month transition probabilities and the average costs) from an observational data of patients who received care and treatment at Arba Minch hospital HIV clinic. The detail is given below.

### Study subjects

We collected outpatient service use and corresponding cost and effect data from two prospective cohorts of HIV patients who received care and treatment at Arba Minch Hospital from January 2003 to March 2006 [17-19]. The first cohort comprised HIV patients who received care without ART at the HIV clinic from January 2003 to April 2004 (15 months). The second cohort was made up of HIV patients who received ART from August 2003 to March 2006 (31 months). Patients in the no-ART cohort who later went on receiving ART were excluded from the no-ART cohort, but included in the ART cohort. Patients who were under the age of 15 years were excluded from the study. In the no-ART cohort, the proportions of patients in the non-AIDS and AIDS states were 0.89 and 0.11 respectively, and in the ART cohort the proportions were 0.74 and 0.26 in the non-AIDS and AIDS states respectively (Table 1). The inpatient service use and corresponding costs were derived from 58 HIV patients (33 without ART and 25 with ART) who received inpatient care at Arba Minch Hospital during 2004/5 Ethiopian fiscal year.

**Table 1:** Profile of study subjects

**Variables**	**Category**	**no-ART**	**ART**
		
		**n(%)**	**n(%)**
**Gender**			
	Female	98(48.3)	88(42.1)
	Male	105(51.7)	121(57.9)
**Age group**			
	<20	7(3.4)	3(1.4)
	20–29	64(31.5)	56(26.8)
	30–39	92(45.3)	90(43.1)
	40–49	30(14.8)	46(22.0)
	>49	10(4.9)	14(6.7)
**WHO Clinical Stages**			
	I	22(10.8)	
	II	30(14.8)	15(7.2)
	III	129(63.6)	139(66.5)
	IV	22(10.8)	55(26.3)
**AIDS status**			
	non AIDS	181(89.2)	154(73.7)
	AIDS	22(10.8)	55(26.3)

	**Total**	**203(100)**	**209(100)**

### Clinical data

The primary clinical outcome of interest was LYG with and without ART. We collected the following data from the patients' records: demographic characteristics, date of starting treatment, clinical stage upon enrolment, presence of opportunistic infections upon enrolment, investigation performed, drugs used, frequency of outpatient visit to the HIV clinic, date of progression to another clinical stage, and time of discontinuing care at the HIV clinic (due to death, lost to follow up or starting ART). The HIV clinic at AMH maintain a database of the HIV patients under its care. The community health workers at the clinic verify and confirm all deathes thar occur outside the hospital through regular home visits and followup. Patients who were not reported to have died or transferred but failed to show up within 90 days after their next scheduled visit were considered as lost to follow-up. Patients were censored on the date of their transfer, status of lost to follow-up, starting ART or the last date of observation whichever came first.

We estimated the disease progression probabilities for each reiteration interval of the 3-month period (cycle) using the Kaplan Meier method. For each cycle we calculated the hazard rates and converted them to the corresponding transition probabilities and their 95% confidence intervals. Accordingly, for the no-ART cohort, we arrived at five and four sets of transition probabilities during observation periods of 1.25 and 1 year for the non-AIDS and AIDS states, respectively. In the ART cohort, we estimated eleven sets of transition probabilities for both the non-AIDS and AIDS states over the observation period. Tables 2 and 3 report the three month transition probabilities observed during the follow up period. We used the average of these probabilities to construct matrices of constant transition probabilities (Table 4), which we applied to all cycles in the respective no-ART and ART scenarios in our model.

**Table 2:** Three-month transition probabilities (95% CI) to a dead state from the non-AIDS and AIDS states among HIV patients at Arba Minch Hospital.

**Time interval (in months)**	**no-ART**		**ART**	
		
	**non-AIDS**	**AIDS**	**non-AIDS**	**AIDS**
	
0–3	0.123(0.084–0.177)	0.270(0.143–0.436)	0.099(0.063–0.151)	0.319(0.237–0.409)
3 to 6	0.123(0.099–0.146)	0.178(0.116–0.196)	0.040(0.032–0.046)	0.021(0.018–0.022)
6 to 9	0.076(0.060–0.091)	0.093(0.074–0.108)	0.032(0.026–0.037)	0.046(0.040–0.048)
9 to 12	0.123(0.093–0.149)	0	0.018(0.015–0.022)	0
12 to 15	0		0	0
15 to 18			0.013(0.010–0.017)	0
18 to 21			0	0
21 to 24			0	0
24 to 27			0	0
27 to 30			0	0
30 to 33			0	00
Average of All quarters	0.090(0.068–0.115)	0.141(0.085–0.194)	0.019(0.013–0.027)	0.040(0.029–0.053)

**Table 3:** Three-month probabilities (95%CI) of progression from non-AIDS to AIDS state among HIV patients at Arba Minch Hospital.

**Time interval in month**	**no-ART**	**ART**
0–3	0	0
3 to 6	0.073(0.034–0.150)	0.015(0.004–0.058)
6 to 9	0.043(0.025–0.068)	0.00856(0.004–0.013)
9 to 12	0	0
12 to 15	0.077(0.024–0.182)	0
15 to 18		0
18 to 21		0
21 to 24		0
24 to 27		0
27 to 30		0
30 to 33		0
Average of all quarters	0.039(0.017–0.083)	0.002(0.001–0.007)

**Table 4:** Three month average transition probabilities matrices (no-ART and ART)

**no-ART**				**ART**			
	
	Transition to				Transition to		
			
Transition from	non-AIDS state	AIDS state	Death	Transition from	non-AIDS state	AIDS state	Death
	
non-AIDS state	0.87	0.04	0.09	non-AIDS state	0.978	0.002	0.02
AIDS state	0	0.86	0.14	AIDS state	0	0.96	0.04
Death	0	0	1	Death	0	0	1

### Cost data

The costing in our study was done from the health care provider's perspective and included both outpatient and inpatient hospital costs. Costs were estimated for both direct capital and recurrent inputs for the final HIV-related services, and their shares from the overhead cost centres of the hospital were also included. The Ethiopian fiscal year starting 8 July 2004 and ending 7 July 2005 was used as the base year, and cost data were collected retrospectively.

We retrieved personnel cost and unit costs of each of the non-medical supplies recurrent inputs from the financial records of the accounts section of AMH. For capital inputs, we took the 2005/6 replacement price from the market. We obtained the 2004/5 price of drugs and medical supplies from the Pharmaceuticals and Medical Supplies Import and Wholesale Share Company (PHARMID) and the SNNPR Health Bureau. We used 2005/6 prices if 2004/5 prices were not available. We allocated hospital overhead costs to the HIV-related services using a stepping-down approach [15]. We used the volume of service during the base year (2004/5) for calculating unit costs of services, and translated the 2005/6 costs to the base year 2004/5. Average annual outpatient and inpatient costs and the respective 95% confidence intervals were estimated from the annual cost of the HIV/AIDS health care service at the hospital. The details of the costing are discussed elsewhere [13]. All costs were converted to the US dollar using the average exchange rate in 2004/5 (US$1= ETB 8.6649) [20].

Then we calculated the three month (i.e. Markov cycle) average cost (Table 5) for each of the Markov 'alive' states (i.e. non-AIDS and AIDS) for both the no-ART and ART scenarios from the respective annual average costs.

**Table 5:** Average three-month (Markov cycle) cost (95% CI) of HIV-related services in US$ * at Arba Minch Hospital by ART and AIDS status.

**Categories**	**no-ART**		**ART**	
	
	**non-AIDS**	**AIDS**	**non-AIDS**	**AIDS**
**Outpatient services**				
***HIV clinic consultation***	3.37(3.09–3.65)	4.41(2.91–5.85)	8.48(8.19–8.77)	8.43(7.86–9.00)
***Laboratory***	2.54(2.28–2.79)	3.20(1.87–4.54)	3.37(3.17–3.58)	3.59(3.12–4.06)
***Imaging***	0.72(0.54–0.90)	1.81(0.70–2.93)	0.11(0.05–0.16)	0.08(0.01–0.17)
***Anti retroviral drugs***	0.00	0.00	45.98(41.40–50.57)	45.67(35.22–56.12)
***Non-Anti-retroviral drugs***	2.50(1.89–3.11)	5.60–(1.94–9.24)	0.97(0.76–1.17)	0.88(0.64–1.12)
				
***Total three months cost of*** ***Outpatient services***	9.12(8.20–10.04)	15.02(8.88–21.16)	58.91(54.11–63.70)	58.65(48.12–69.19)
				
**Inpatient services:**				
***Laboratory***	0.81(0.52–1.09)	0.86(0.63–1.08)	0.83(0.52–1.13)	0.60(0.09–1.10)
***Imaging***	0.43(-0.01–0.88)	0.68(0.36–0.99)	0.06(-0.7–0.18)	0.19(-0.29–0.67)
***Non-Anti-retroviral drugs***	2.81(1.93–3.68)	2.85(1.89–3.80)	1.41(0.93–1.90)	1.24(0.23–2.25)
***General Care & treatment***	8.31(4.27–12.35)	14.43(10.21–18.65)	3.09(2.40–3.79)	6.27(3.89–8.64)
***Meal***	2.74(1.41–4.07)	4.75(3.36–6.14)	1.02(0.79–1.25)	2.06(1.28–2.85)
				
***Total three months cost of*** ***in-patient care***	15.10(8.81–21.38)	23.55(17.81–29.29)	6.41(5.23–7.60)	10.35(6.94–13.77)

* US$1 = 8.6649 Ethiopian birr

### Base case cost-effectiveness analysis

At base case analysis, we populated the model with the initial distribution patients in the no-ART and ART scenarios as observed from the Arba Minch cohort (Table 1), the three month average transition probabilities (Table 4) and the three month average costs (Table 5). we assumed that ART would have a continuous treatment effect and the survival divergence observed [18] during the first few cycles would continue throughout the remaining cycles. The survival pattern between the no-ART and ART cohorts at AMH showed that, after the first few months of follow up, the proportion surviving in the ART group became higher than that of the no-ART group and remained higher throughout the observation period. Thus, assuming the same survival pattern would also continue after the follow up, we applied the average of the transition probabilities observed during the follow-up period to all cycles in our model. The model is then run for 80 cycles (20 years) for a hypothetical cohort of patients to estimate the life expectancies and life time costs.

We present the results in terms of incremental costs, incremental LYG and incremental cost per LYG [15], first undiscounted and then at a 3 % discount rate. We used the criteria outlined in the World Health Report 2002 [21] to assess whether ART is a cost effective intervention in the Ethiopian setting. The report indicates those interventions with an incremental cost effectiveness ratio (ICER) within the value three times the per capita GDP as cost effective; and those interventions with ICERs less than the per capita GDP as highly cost effective.

### Handling of uncertainties

We used probabilistic sensitivity analysis (PSA) to assess the overall impact on the results from simultaneous uncertainties around the input parameters, and present the result in the form of a cost-effectiveness acceptability curve (CEAC) [15]. The CEAC illustrates the probability that ART is cost effective for different levels of willingness of the health care provider to pay for LYG, which is likely to depend on the availability of resources for health care in different settings.

To deal with uncertainties related to the model structure and process, we performed scenario analysis under the following conditions: (a) if the initial distribution of patients in the non-AIDS and AIDS Markov states is changed and all patients start from the non-AIDS Markov state; (b) if the time horizon of the model evaluation is extended and the model analysis continue until all the patients in the ART cohort die; (c) if different amounts of one time additional costs are included for patients dying under the ART scenario; and (d) if effect of ART is limited to cycles that correspond to the observation period and has no effect thereafter (i.e. Treatment effect only during the first eleven cycles).

### Statistical tools and ethical considerations

We used a CostIt version 4.4 spreadsheet [22] to categorize and summarize the cost data, SPSS version 14.1 and Stata version 9.2 for the statistical analysis of patient level data and TreeAge software (Pro 2005 suite version 1.4) for the cost-effectiveness analysis. Ethical clearance and permission to access hospital records were obtained from the Southern Nation Nationalities and Peoples Region Health Bureau. Patients also have given individual written consent before enrolment into the study [18].

## Results

### Profile of study subjects

#### No-ART cohort

Two hundred and nine adult HIV patients visited the HIV clinic at Arba Minch Hospital from January 01, 2003 to April 08, 2004 (15.2 months). Five of these patients had incomplete records and one patient died on the day of the first visit. These patients were therefore excluded from the study. The remaining 203 (98 women and 105 men) comprised the no-ART cohort. Among these, 181 patients (89.2%) were non-AIDS (WHO clinical stages I, II and III) and 22 patients (10.8%) had AIDS (WHO clinical stage IV) (Table 1). During the follow-up period 53 patients (26.1%) died; 1 patient (0.5%) was transferred; 31 patients (15.3%) were lost to follow-up; 45 patients (22.2%) were switched to ART; and 73 patients (36%) were under care until the last date of the follow up. The median follow-up period was 14.6 weeks. The mean age of the cohort was 32.6 years and the average monthly income was US$23.43.

#### ART cohort

Two hundred and ten adult HIV ART patients visited the HIV clinic from August 01, 2003 to March 09, 2006 (31.2 months). One patient had incomplete records, and was excluded from the study. Thus, 209 patients (88 women and 121 men) comprised the ART cohort. Out of these, 154 patients (73.7%) were in the non-AIDS stages and 55 patients (26.3%) had AIDS (Table 1). During the follow-up period 53 patients (25.4%) died; 13 patients (6.2%) were transferred to other ART sites that were close to where the patients lived; 21 patients (10%) were lost to follow-up; and 122 patients (58.4%) were under care until the last date of the follow up. The median follow up period was 49.14 weeks. The mean age was 34.4 years and the average monthly income was US$30.12.

### Base case analysis

#### Expected survival and lifetime cost

For the no-ART scenario, the undiscounted expected survival in the non-AIDS and AIDS states were 1.6 and 0.7 years, respectively, and the total life expectancy was 2.3 years. The expected total lifetime cost of care to HIV patients without ART was US$265 and the average PPY cost is US$114 (Table 6).

**Table 6:** Base case cost-effectiveness (95% CI) of ART compared with no-ART

**Strategy**	**Life time Cost $**	**Life time incremental cost$**	**Life years gain**	**Incremental life years gain**	**Cost effectiveness ($)**	**Incremental Cost Effectiveness ratio (ICER $)**	**% change from base case ICER**
***Undiscounted***							
no-ART	265(86–1148)		2.3(1.95–2.73)		114(38–504)		
ART	2479(1214–4828)	2215(771–4635)	9.4(8.67–10.05)	7.1(6.24–7.83)	265(130–513)	314(111–654)	
							
***Discounted at 3%***							
no-ART	247(80–1008)		2.2(1.83–2.56)		114(37–471)		
ART	2028(1000–3868)	1780(577–3710)	7.7(7.15–8.18)	5.5(4.85–6.13)	265(131–508)	325(106–680)	3.5

For ART, at base case, the total undiscounted life expectancy was 9.4 years. The respective survival times before progressing to the next worst state in the non-AIDS and AIDS states were 7.3 and 2.1 years, respectively. The expected lifetime cost of treating patients with ART was US$2,479 with an average cost of US$265 PPY.

### Incremental costs and cost-effectiveness

The cost-effectiveness analysis showed that ART is both a more costly and more effective scenario compared with the no-ART scenario. ART prolonged the life of patients by an average of 7.1 years with an additional lifetime cost of US$ 2,215, resulting in an average undiscounted incremental cost-effectiveness ratio (ICER) of US$314 per LYG (Table 6). The ICER represents the additional cost needed over the cost of no-ART to extend life expectancy by one more year. When both costs and effects are discounted at 3%, the average extra LYG decreases to 5.5; and the average incremental cost per additional LYG (i.e. ICER) increases to US$325. (Table 6)

### Uncertainties

#### Parameter uncertainty

**Figure 2:**
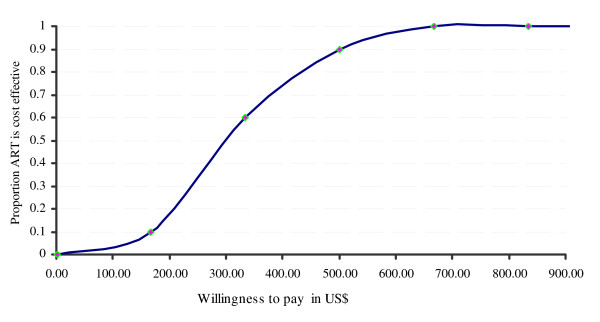
**Cost-effectiveness acceptability curve for ART**.

Figure 2 summarizes the probabilistic sensitivity analysis (PSA) results. The cost-effectiveness acceptability curve suggests the probability of ART being cost-effective for different levels of willingness to pay for a LYG. Accordingly, the probability that ART will be cost effective is about 85% given the level of willingness to pay as approximated by the value 3 times the base year per capita GDP of US$169 [23] in Ethiopia (i.e. US$507).

#### Scenario analysis and comparison with base case cost-effectiveness

Table 7 summarizes the results of the scenario analysis. At base case, patients were distributed into the non-AIDS and AIDS Markov states as observed from the Arba Minch Hospital cohorts. Under the differing assumption that all the patients in each cohort were initially in the non-AIDS state, the Markov cohort analysis resulted in an average of 8 LYG with an average incremental cost of US$307 per LYG (Table 7-a) compared to the averages 7.1 extra LYG and US$314 incremental cost in the base case result.

**Table 7:** Cost-effectiveness (95% CI) of ART compared to no-ART under different scenarios

**Strategy**	**Life time Cost $**	**Life time incremental cost**	**Life years gain**	**Incremental life years gain**	**Cost effectiveness ($)**	**Incremental Cost Effectiveness ratio (ICER $)**	**% change from base case ICER**
***a)***							
no-ART	264(82–1046)		2.4(1.99–2.82)		110(36–433)		
ART	2713(1038–5883)	2448(653–5665)	10.4(9.45–11.23)	8(6.96–8.91)	262(101–564)	307(83–708)	-2.2
							
***b)***							
no-ART	265(85–1148)		2.3(1.95–2.73)		114(38–504)		
ART	3022(1486–6054)	2757(1080–5885)	11.4(10.46–12.41)	9.1(8.03–10.18)	264(129–525)	303(121–634)	-3.5
							
***c)***							
no-ART	265(85–1148)		2.3(1.95–2.73)		114(38–504)		
ART*	2537(1281–4859)	2273(851–4647)	9.4(8.67–10.05)	7.1(6.24–7.83)	271(137–519)	322(120–662)	2.6
ART**	2576(1313–4937)	2311(844–4740)	9.4(8.67–10.05)	7.1(6.24–7.83)	275(141–525_	328(123–668)	4.5
ART***	2672(1413–5069)	2407(1020–4841)	9.4(8.67–10.05)	7.1(6.24–7.83)	285(151–539)	341(145–684)	8.6
							
*d)*							
no-ART	265(85–1148)		2.3(1.95–2.73)		114(38–504)		
ART	1150(621–2099)	886(-100–1924)	4.3(3.99–4.67)	2.0(1.49–2.53)	266(145–482)	441(-49–1009)	40.5

a) if all patients initially start from the non-AIDS state

b) if the model runs until all the patents in the ART cohort die

c) if different amounts of one time additional costs are included for patients dying under the ART scenario

d) if effect of ART is limited to cycles that correspond to the observation period and has no effect thereafter.

(i.e. Treatment effect only for the first 11 cycles)

* if additional cost for dying patients = $70

** if additional cost for dying patients = $117

*** if additional cost for dying patients = $233

Regarding the time horizon, the base case analysis was based on evaluation of the model for 80 cycles (i.e. 20 years); and a significant proportion of the ART hypothetical cohort was alive when the model stopped running. In the alternative scenario, we removed the 20-year assumption and ran the model until all the patients in the cohort would die. This resulted in increased life expectancy, increased lifetime cost and a 3.5% reduction in the average incremental cost per LYG or the ICER (Table 7-b).

The pattern of service use and corresponding costs, in our study, would be higher in the no-ART scenario if the costs of ARV were removed from the ART scenario. In the base case analysis we assumed that this difference might implicitly reflect that the cost of dying was captured in both the no-ART and ART wings. To assess the sensitivity of this assumption and its potential effect on the base case ICER, we analyzed the model under three different scenarios of additional cost of dying for patients dying under the ART scenario. We used additional dying costs of $70, $117 and $233, which were respectively the three, five and ten times the three month average cost for AIDS patients under ART, which was US$ 23.3 excluding the cost of ARV drugs. This resulted in increased per LYG average costs (i.e. ICERs) that range from US$322 to US$341 with a maximum of 9% increase from the base case ICER (Table 7-c).

While the alternative scenarios described above (Table 7a–c) had only relatively small effects on the cost effectiveness of ART, the impact of a pessimistic assumption about future treatment effect was more substantial. At base case, we assumed a continuous treatment effect of ART. In contrast to this, in an alternative scenario, we assumed that ART would have no effect after the actual follow-up period. Thus, we applied the average transition probabilities of the no-ART scenario to the ART scenario after the eleventh Markov cycle (i.e. for Markov cycles beyond the follow-up period). Under this scenario, while the base case incremental cost per LYG (i.e. the ICER) increases by 41%, the additional LYG and corresponding additional lifetime cost decrease by 72% and 60% respectively (Table 7-d). The probability that ART would be cost-effective also decreases to 58%.

## Discussion

### Principal findings

At base case, ART resulted in 7.1 extra LYG with lifetime incremental cost of US$2,215 and undiscounted incremental cost per LYG of US$314. However, the extra LYG increases to 8 years and the incremental cost PPY reduces to US$307 if the ART is started while the patients are in the non-AIDS state (i.e. WHO clinical stages I–III) and before progressing to the AIDS state (WHO clinical stage IV). The result is sensitive to assumptions about the extrapolation of the treatment effects of ART, but stable to discounting, extending the time span of the model evaluation and the inclusion of separate cost of dying.

There are no benchmark values for cost-effectiveness in the Ethiopian context. However ART appears cost effective when compared with the threshold suggested in the World Health Report 2002 [21]. The report suggests that interventions costing less than per capita GDP per LYG are highly cost effective, and interventions costing less than three times the per capita GDP are cost effective Though the incremental cost per LYG under the assumption of reducing treatment effect of ART was considerably higher than that of the base case, all the incremental costs per LYG are less than three times the per capita GDP (US$169) at the base year [23], i.e. US$507 (Tables 6 and 7).

### Discussion of main findings

The incremental LYG through ART are fewer in our study than has been reported from South Africa by Cleary et al [7], who reported 10 undiscounted and 6.8 discounted incremental life years through ART. Though there could be several reasons for these differences, one reason for observing fewer extra LYG in our study could be the time horizon used in our model. We set the time horizon of our study to 20 years (80 cycles). The alternative scenario of running the model until all the patients in each cohort die would yield 9.1 extra undiscounted LYG with ART, which is comparable to the South African study.

While the base case LYG in our study was smaller than previously modelled, the incremental cost-effectiveness appears more favourable than previously observed [4,7,9]. When the incremental costs are adjusted to a common year (i.e. 2005 US$), the estimate from Cote d'Iviore ranges from US$574 to US$874 and that of South Africa ranges from US$1,095 [7] to US$1,868 [4]. One should, however, be careful in making direct comparison of the cost-effectiveness values of different settings due to varied assumptions and contexts [24]. Nevertheless, the lower prices of labour and non-traded inputs in Ethiopia might have lowered the cost estimates.

### Study limitations

We categorized the course of HIV into the Markov states based on the WHO clinical staging system [12], which is commonly applied in settings where immunological and virological measures are not available. However, as the Markov non-AIDS state included 3 distinct stages (i.e. WHO clinical stages I–III) with assumable varied risk of progression, ideally it might have been better to have applied a Markov model with the corresponding number of health states. This was, however, not feasible in our case because of limited data. In addition, in our model, the initial proportion of patients in the AIDS state under ART was higher than those under no-ART. This might have introduced bias as those patients in the AIDS state are likely to have higher mortality. The higher proportion of AIDS patients under ART was due to the fact that when the ART program was initiated at AMH, there were already a considerable number of AIDS cases and priority was given to those patients thereby increasing the number of AIDS cases under ART. The model corresponds well with the nature of the empirical evidence.

As mentioned, we also assumed a continuous treatment effect of ART and applied the average transitional probabilities observed during the follow-up period to all the Markov cycles. The sensitivity analysis indicated that making different assumptions about this has relatively large implications for cost-effectiveness estimates. The reason for assuming a continuous treatment effect of ART was that the survival pattern along the no-ART and ART cohorts at Arba Minch Hospital showed that the short-term survival curves of the cohorts diverged within the first 6-month observation and continued to diverge even more as the mortality under ART tended to stabilize [18]. Nevertheless, there are indications that treatment side effects and drug resistance are possibilities in the long course of ART [25-27]. Such undesirable outcomes have the potential to dilute the treatment effect in the long run and may eventually result in an increased disease progression even under treatment, resulting in increased cost. From this perspective, our assumption of continuous treatment effect might have underestimated the disease progression under the ART scenario in the long run and overestimated the corresponding survival. The alternative scenario where the effectiveness of ART was assumed to decrease after the follow-up period resulted in reduced LYG and higher incremental cost per LYG (Table 7-d). However, the assumption that ART will not have an effect after the observation period is an extreme scenario and might be unlikely. Though the mortality of HIV patients treated with ART is still higher than that of the general population in the sub-Saharan Africa, there is an indication that it is becoming comparable to that of the general population as the treatment continues [28].

Another potential limitation is that we measured the health outcome in terms of LYG. This is a straight forward choice of method, but imposes some restrictions on the generalizability of the results. Our method provides useful information on life expectancies, but does not capture the morbidity dimension of the health improvement and, therefore, does not reveal the full benefit of ART [29,30]. As HIV/AIDS is a life long problem, incorporating the health improvements of ART using Quality Adjusted Life Years (QALY) would have enriched the results. However, no quality of life weights for HIV patients in Ethiopia are available. In the choice between using disability weights (DALYs) with questionable validity for countries such as Ethiopia or the less commensurable LYG [29], we chose the latter approach. The choice of LYG as a measure of health benefits may have biased our estimates conservatively.

The identification, measurement and valuation of the costs followed an ingredient approach. Most of the costs associated with the HIV-related services and overhead activities were, therefore, included in the cost estimation. Nevertheless, there are few uncertainties and shortcomings in our costing approach. First, we applied average rather than marginal unit costs in the cost analysis, which is a problem if the unit costs are much affected by the volume of service delivered during a specified period [15]. Service categories that operate below their full capacity, for example, are likely to have higher average rather than marginal costs, while the marginal costs of services operating above the limit of their capacity are likely to be higher than the average costs. Second, due to the difficulty in retrieving retrospective inpatient service use data for HIV patients at Arba Minch hospital, we based the estimate of inpatient costs on a single year service use data which may not fully reflect the pattern of use of service over a number of years.

The assessment of cost effectiveness relative to GDP thresholds assumes both societal costs and gains. Taking only the health care provider's perspective may not reflect the full societal costs. Therefore, the omission of logistic costs and patient-incurred costs, such as transport costs and lost productivity due to care seeking, might have the potential to make the ART appear more cost-effective than it actually is. On the other side we excluded patient level indirect costs including productivity changes. ART often improves patients' health to a level that enables them to resume their usual subsistence activities. These effects, if included, might have pulled the cost effectiveness in a more favourable direction. We believe our costing perspective is conservative.

## Conclusion

The estimated annual average cost has direct implications for program management. In rural district hospital settings, Ethiopia would need to spend about US$265 per patient per year to deliver ART. This may help in planning, budgeting and financing for expanding ART in Ethiopia. Nevertheless, though there may be no major differences in the epidemiology of HIV and prices of major inputs across the country, the cost drivers are likely to be different across levels of hospitals. Therefore, our cost findings may not reflect the pattern in the relatively cosmopolitan centres where major tertiary hospitals operate. However, as Arba Minch Hospital is a typical district hospital in Ethiopia, our findings may be extended to other district hospital settings in the country.

Our study is the first economic evaluation of ART in an Ethiopian context and may have implications for scaling up AIDS treatment in the country. According to the WHO guideline [21], ART in Ethiopia appears cost effective, though may not be regarded as highly cost effective. The incremental cost per additional LYG is below three times the per capita GDP, but it is above the per capita GDP, implying a need for more economically efficient ways of delivering ART. This may involve investigating: (i) whether providing ART through integrated service is preferable to specialized clinics, and (ii) the cost-effectiveness of delivering ART through health centres compared to hospitals.

## Competing interests

The authors declare that they have no competing interests.

## Authors' contributions

ADB designed the study, analyzed the data and wrote the manuscript. DJ established the Arba Minch Hospital HIV cohort database and contributed in editing the manuscript. BR contributed to the design of the study, analysis of the data, and writing of the manuscript. BL contributed to the conception and design of the study, analysis of the data, and writing of the manuscript.
